# Anton-Babinski Syndrome in a Recurrent Ischemic Stroke Patient: A Case Report

**DOI:** 10.7759/cureus.44599

**Published:** 2023-09-03

**Authors:** Mei Yin Pong, Jun Fai Yap, Tze Yang Chung, Soo Chin Chan, Sakinah Sabirin

**Affiliations:** 1 Rehabilitation Medicine, Universiti Malaya, Kuala Lumpur, MYS; 2 Rehabilitation Medicine, University Malaya Medical Centre, Kuala Lumpur, MYS; 3 Social and Preventive Medicine, Universiti Malaya, Kuala Lumpur, MYS; 4 Public Health, University Malaya Medical Centre, Kuala Lumpur, MYS

**Keywords:** malaysia, cortical blindness, cerebrovascular accident, confabulation, visual anosognosia

## Abstract

Anton-Babinski syndrome (ABS) is a rare neuropsychiatric condition characterized by visual anosognosia (denial of vision loss) and confabulation in the presence of intact anterior visual tracts. The most common cause of ABS is a cerebrovascular accident involving bilateral occipital lobe injuries with varying degrees of cortical blindness. In this report, we present the case of a woman with suspected ABS following a recurrent ischemic stroke in Malaysia. Establishing a proper diagnosis of stroke is crucial for modifying rehabilitation goals to ensure improved functional outcomes.

## Introduction

Anton-Babinski syndrome (ABS) refers to an uncommon neuropsychiatric syndrome featuring visual anosognosia (denial of vision loss) associated with confabulation in cortically blind patients. A cerebrovascular accident involving bilateral occipital lobes with different degrees of cortical blindness is one of the several causes [1]. The exact pathophysiology of ABS remains unclear. Generally, damage to both the primary visual cortex (bilateral occipital lobes) and visual association areas (temporal and parietal lobes) can happen in ABS. Visual anosognosia may arise from a disconnection between the white matter of the parietal lobe and functioning areas such as speech and language areas. This lack of input leads to confabulation as the ABS patient attempts to fill in the missing sensory information [2]. We present the case of a Malaysian woman with suspected ABS following a recurrent ischemic stroke. 

## Case presentation

A 50-year-old Malay woman with underlying hypertension, type II diabetes mellitus, and dyslipidemia had a recurrent ischemic stroke, characterized by severe headache, vomiting, and sudden onset of blurred vision. For her first ischemic stroke episode, which occurred five years ago, she suffered from left hemiparesis and left homonymous hemianopia. Following intensive rehabilitation, she recovered without residual weakness. However, she continued to experience visual impairment that affected her work performance and subsequently stopped taking her medications for secondary stroke prevention.

Unlike her first stroke, there was no limb weakness or sensory impairment during her recurrent ischemic stroke this time. Despite clear visual impairments, evidenced by her frequent collisions with objects while walking or using furniture for ambulation, the patient denied experiencing any visual loss. Additionally, she exhibited confabulation, adamantly claiming to have completed household chores that had not been done. On examination, her visual acuity, tested via objective refraction, was bilateral hand movement. Her eye tracking and light perception were preserved, though her visual field loss using the confrontation method yielded inconsistent results. Nonetheless, her anterior segment and fundoscopic examination showed normal findings.

**Figure 1:**
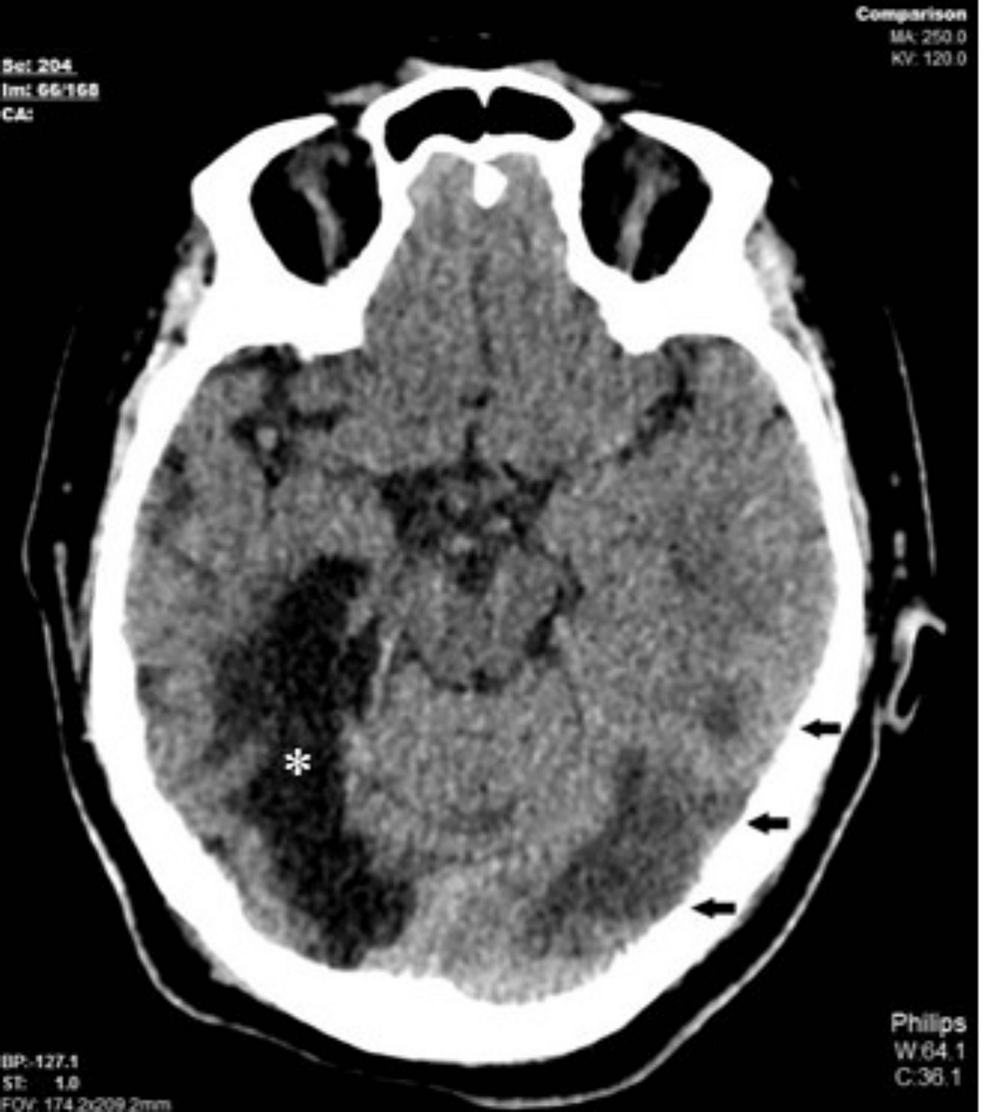
CT of the brain in the axial plane showed ill-defined hypodensities in the left occipital lobe (black arrows), consistent with a recent infarct. Another well-defined hypodensity was noted in the right parieto-occipital-temporal lobe, along with ex vacuo dilatation of the temporal horn of the right lateral ventricle, in keeping with encephalomalacic changes due to a previous right PCA territory infarct (*). CT: computed tomography; PCA: posterior cerebral artery.

**Figure 2:**
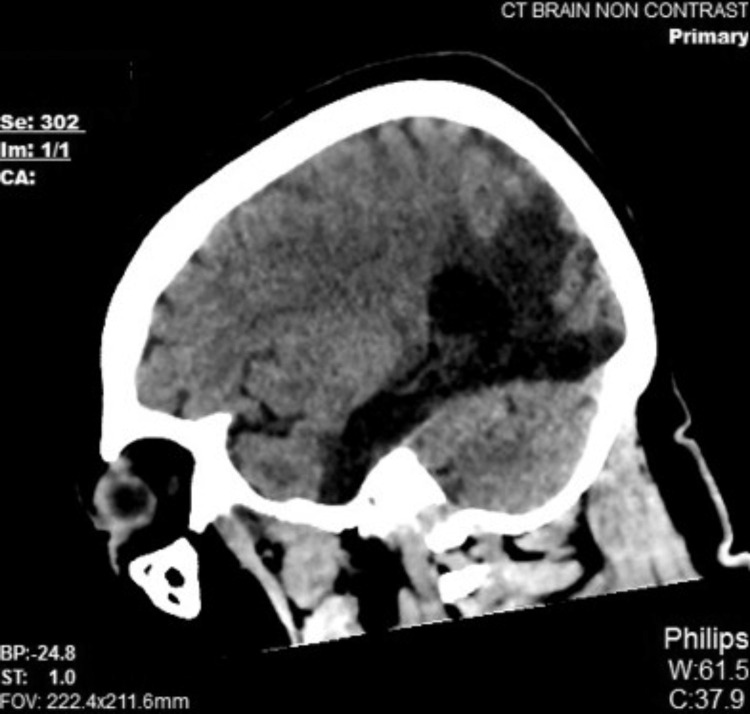
CT of the brain in the sagittal plane (multiplanar reconstruction) demonstrated the extent of the encephalomalacic changes. CT: computed tomography.

Computed tomography angiography (CTA) of the cerebral arteries revealed subacute hypodensities in the left occipital lobes and a well-defined hypodensity in the right parieto-occipital-temporal lobe, along with ex vacuo dilatation of temporal horn of the right lateral ventricle, in keeping with encephalomalacia changes (Figures 1, 2).

**Figure 3:**
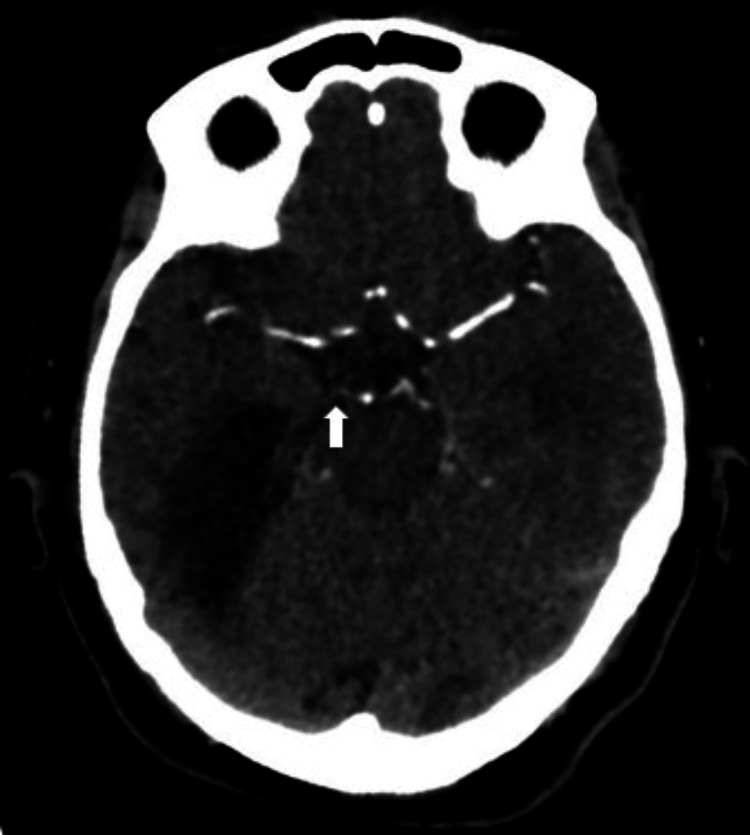
Cerebral CTA in the axial plane showed bilateral PCA stenoses (right > left) at the level of the circle of Willis. Small caliber and faint opacification were noted in the P1 segment of the right PCA (white arrow). CTA: computed tomography angiography; PCA: posterior cerebral artery.

**Figure 4:**
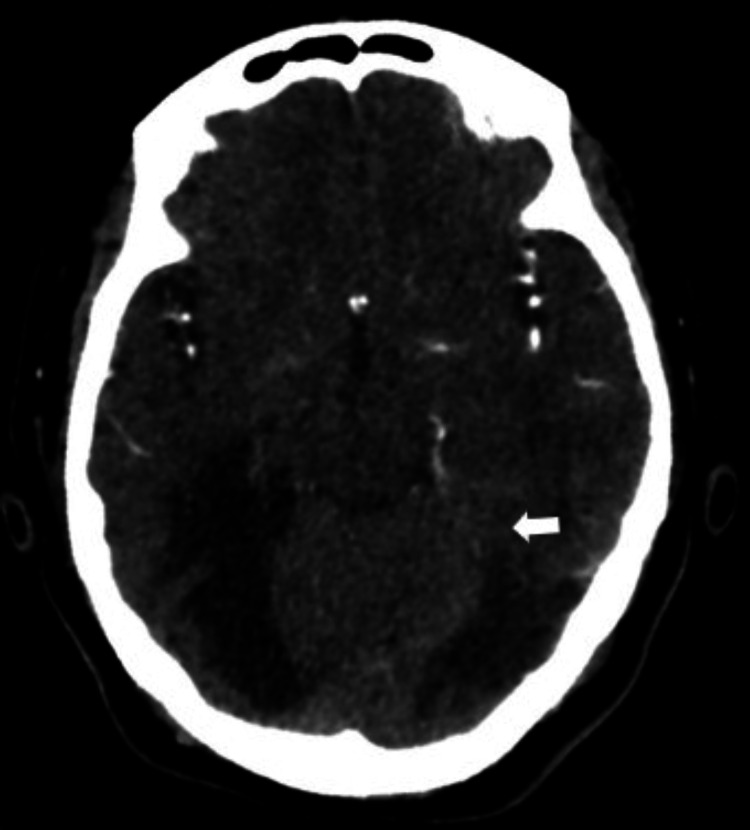
Cerebral CTA in the axial plane showed reduced opacification over the P4 segment of the left PCA (white arrow). CTA: computed tomography angiography; PCA: posterior cerebral artery.

Mild-to-moderate stenoses were noted in the bilateral posterior cerebral artery (PCA) (Figure 3), along with dense calcification of the right vertebral artery and reduced opacification over the P4 segment of the left PCA (Figure 4).

**Figure 5:**
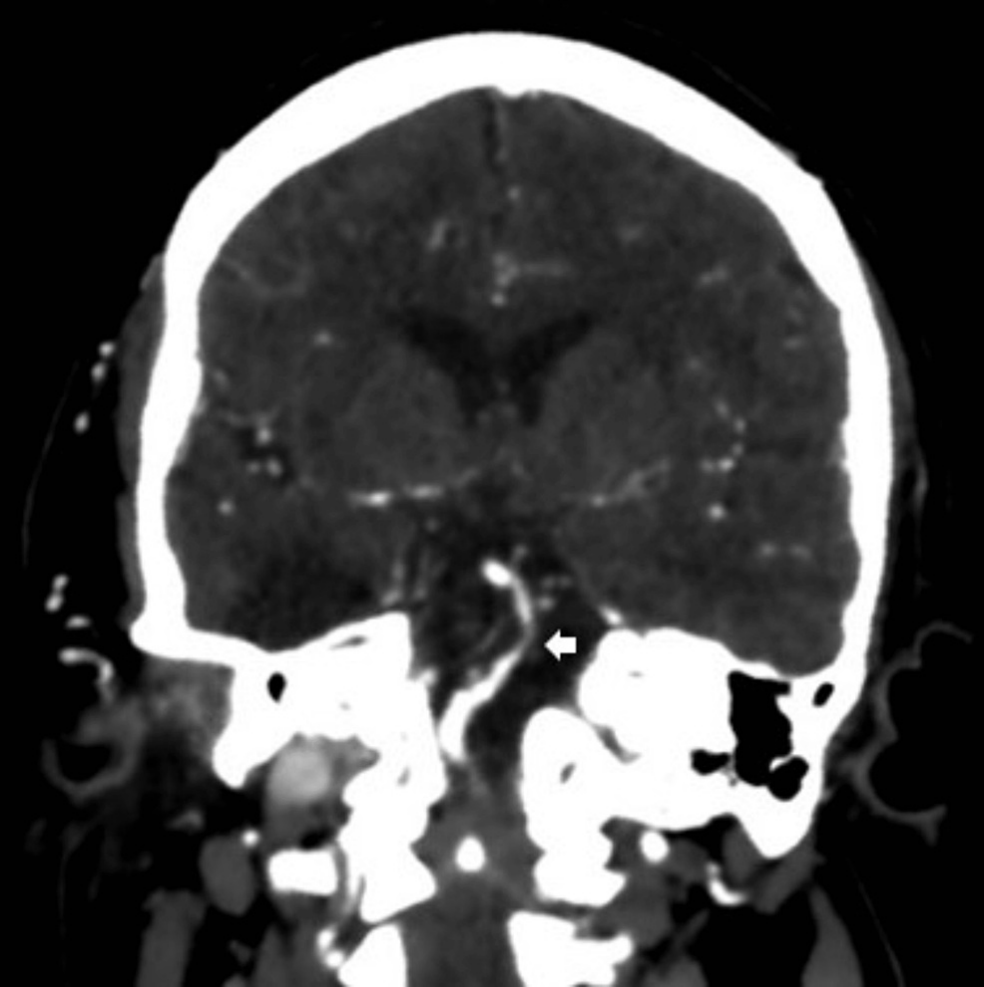
Cerebral CTA in an oblique coronal view showed severe stenosis at the middle part of the basilar artery (white arrow). CTA: computed tomography angiography.

**Figure 6:**
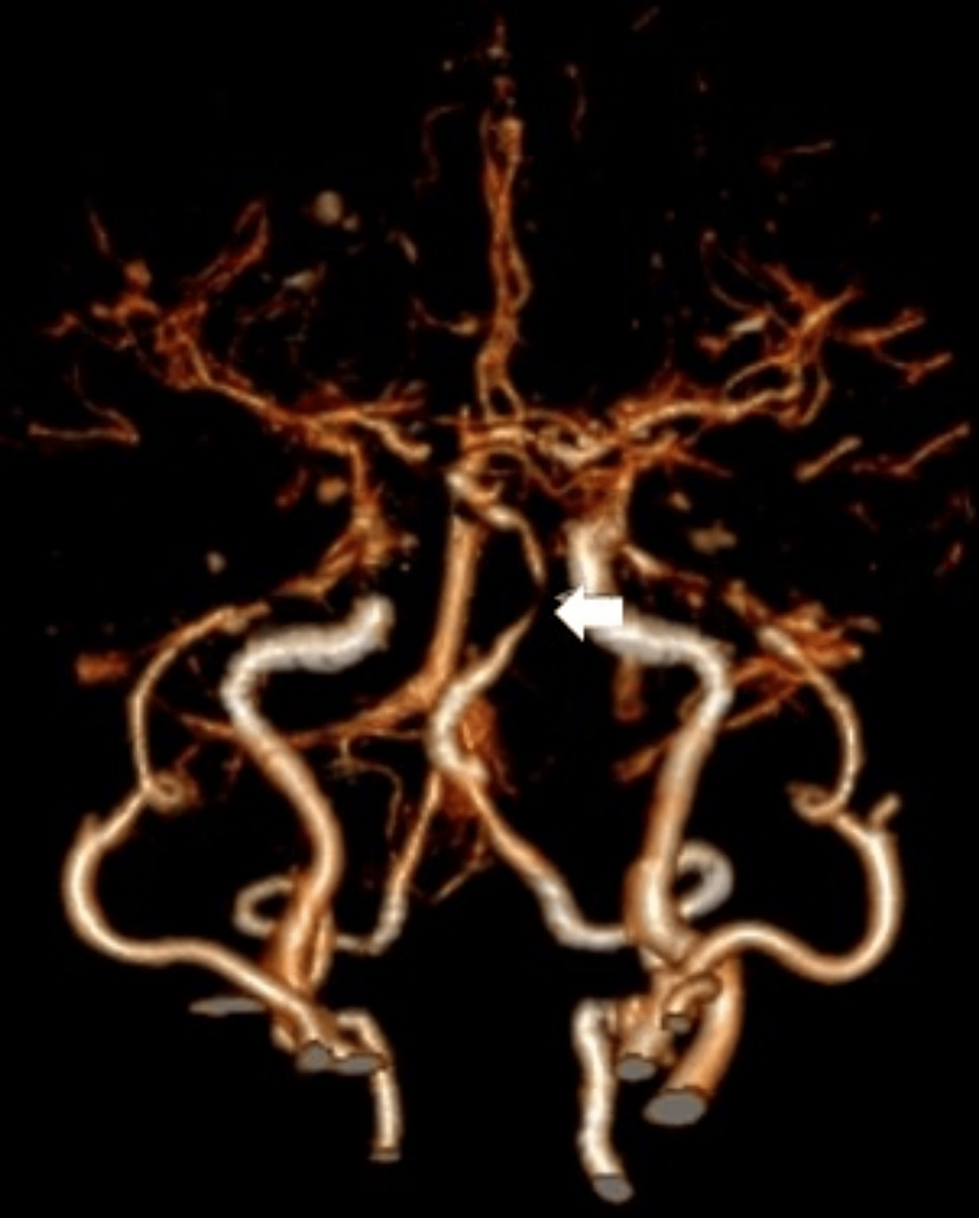
Cerebral CTA (volume rendering technique using maximal intensity projection) showed severe stenosis at the middle part of the basilar artery (white arrow). CTA: computed tomography angiography.

Both cerebral CTA in oblique coronal view and volume rendering technique using maximal intensity projection showed severe stenosis at the middle part of the basilar artery (white arrow) (Figures 5, 6).

Clinical and radiological features supported a diagnosis of ABS. The patient had a right PCA infarct with left homonymous hemianopia prior to the subacute left PCA infarct. Remarkably, her visual anosognosia was only evident after the second stroke episode. Following the recurrent ischemic stroke, her functional status significantly declined, and intensive inpatient rehabilitation was offered. However, her family members were not keen to accept the offer, prompting the need for 24-hour supervision from family members to manage medications, finances, self-care, and mobility at home. Although she continued to visit the hospital for her scheduled outpatient rehabilitation, her prognosis remained poor due to a lack of an intensive rehabilitation regime and a lack of insight into her current illness.

## Discussion

The rarity and diverse clinical presentations of ABS present challenges in customizing rehabilitation goals. Patients with ABS lack insight into their impairments, which hinders their participation in rehabilitative therapies and compromises functional outcomes. Stroke rehabilitation generally employs two approaches: compensatory and restorative. The compensatory strategy focuses on adapting or finding alternate ways to perform activities of daily living (ADL). ABS rehabilitation, being challenging, primarily emphasizes compensatory methods through sensory rehabilitation, environmental adaptations, and adaptive devices to enhance ADL performance. However, these approaches do not induce improvement in visual perception [3].

Conversely, the restorative approach (utilizing light stimuli) aims to restore visual perception by repetitively stimulating viable neurons surrounding the damaged cerebral cortex, redirecting visual processing through alternative pathways, or employing a combination of these processes based on the concept of neuroplasticity [2,4]. In essence, a combined implementation of both compensatory and restorative strategies can be pursued, depending on the stroke patient’s potential for recovery. 

Unlike ABS, a patient’s personal insight into the visual deficit is preserved in Charles Bonnet syndrome [5]. There is speculation that it shares a similar pathophysiology with phantom limb syndrome [6]. In this context, sensory deprivation resulting from visual impairment causes visual sensory deafferentation, leading to disinhibition, spontaneous activation of the visual cortices, and subsequent production of false substituted images [7]. Nevertheless, the complex visual hallucinations typically observed in Charles Bonnet syndrome lack obvious psychopathology.

In contrast, Balint syndrome is characterized by bilateral parieto-occipital lobe lesions [8]. This visuospatial disorder manifests as a triad: optic ataxia (difficulty in visually guided reaching out for objects), oculomotor apraxia (difficulty in voluntarily shifting gaze despite functioning extraocular muscles), and simultanagnosia (difficulty in perceiving more than a single object) [9]. In the case of our patient, none of these signs were present, making a differential diagnosis of Balint syndrome less likely.

## Conclusions

In cases where a standard ophthalmological examination yields no significant findings, ABS should be considered for patients who deny visual loss, exhibit unrealistic confabulation, and display evidence of bilateral occipital lobe injuries. Management of ABS patients should prioritize secondary stroke prevention and the adaptation of rehabilitation goals to achieve improved functional outcomes. The limited insight associated with ABS presented significant barriers to post-stroke rehabilitation.
